# GENPPI: standalone software for creating protein interaction networks from genomes

**DOI:** 10.1186/s12859-021-04501-0

**Published:** 2021-12-16

**Authors:** William F. Anjos, Gabriel C. Lanes, Vasco A. Azevedo, Anderson R. Santos

**Affiliations:** 1 Department of Computer Science, Federal University of Uberlândia, Uberlândia, Brazil; 2 Biology Institute, Federal University of Uberlândia, Uberlândia, Brazil; 3 Department of Genetics, Federal University of Minas Gerais, Belo Horizonte, Brazil

**Keywords:** Protein, Interaction, Network, Standalone, Software, Bacteria

## Abstract

**BackGround:**

Bacterial genomes are being deposited into online databases at an increasing rate. Genome annotation represents one of the first efforts to understand organisms and their diseases. Some evolutionary relationships capable of being annotated only from genomes are conserved gene neighbourhoods (CNs), phylogenetic profiles (PPs), and gene fusions. At present, there is no standalone software that enables networks of interactions among proteins to be created using these three evolutionary characteristics with efficient and effective results.

**Results:**

We developed GENPPI software for the ab initio prediction of interaction networks using predicted proteins from a genome. In our case study, we employed 50 genomes of the genus *Corynebacterium*. Based on the PP relationship, GENPPI differentiated genomes between the ovis and equi biovars of the species *Corynebacterium pseudotuberculosis* and created groups among the other species analysed. If we inspected only the CN relationship, we could not entirely separate biovars, only species. Our software GENPPI was determined to be efficient because, for example, it creates interaction networks from the central genomes of 50 species/lineages with an average size of 2200 genes in less than 40 min on a conventional computer. Moreover, the interaction networks that our software creates reflect correct evolutionary relationships between species, which we confirmed with average nucleotide identity analyses. Additionally, this software enables the user to define how he or she intends to explore the PP and CN characteristics through various parameters, enabling the creation of customized interaction networks. For instance, users can set parameters regarding the genus, metagenome, or pangenome. In addition to the parameterization of GENPPI, it is also the user’s choice regarding which set of genomes they are going to study.

**Conclusions:**

GENPPI can help fill the gap concerning the considerable number of novel genomes assembled monthly and our ability to process interaction networks considering the noncore genes for all completed genome versions. With GENPPI, a user dictates how many and how evolutionarily correlated the genomes answer a scientific query.

## Background

The annotation of genomes is an important task to perform after sequencing and assembly. Annotating genomes helps researchers to elucidate the functions of predicted open reading frames (ORFs). In this study, we largely assign an ORF’s potential role according to the sequence similarity of proteins or bases to those of previously characterized counterparts [1]. In addition to functionality, there are other features that can be predicted from ORFs, enabling researchers to annotate a genome from a network’s topological characteristics. If we consider ORFs to be vertices and the relationships as edges, a complex network can be constructed from a genome. This hypothetical network’s available information depends on the number of vertices and the quality and stability of the associated edges [2]. After a trustable network is obtained, researchers can experiment with various close and distant relationships among the vertices. A near relation can be defined by the number of edges directly connected to each vertex or the degree of the connections. The vertex degree immediately helps to characterize the magnitude of the number of connections, facilitating the identification of central or peripheral elements of a network of proteins. A distant relation can be defined by referring to the importance of a set of vertices *V* as the most likely to be traversed when connecting an arbitrary pair of points *u* and *x* in a network. We can consider the vertices possessing such larger probabilities to be essential for communication within the web or betweenness centrality. Also, we can mention several other centrality measures that are of great importance to the analysis of data under a topological perspective, such as PageRank, Bridging Coefficient, Bridging Centrality, Density, and Diameter of a network [3]. In addition to investigating the gene’s topological annotation products as isolated features, we have recently employed a set of notes as input for machine learning (ML) algorithms. ML enables us to utilize a new technique for genome annotation [4]. However, when employing all these topological analyses to characterize a genome, enriching its annotation starts with a trustable protein network. At present, the primary source of genome topological annotations is the Search Tool for the Retrieval of Interacting Genes/Proteins (STRING) database. STRING presents annotation data for more than five thousand genomes spread over a wide range of organisms. Such features as conserved gene neighbourhood, conserved phylogenetic profile, gene fusion, Gene Ontology features (molecular function, process, and localization), coexpression, experiments, and bibliographic evidence are conjugated, creating a probabilistic strength of belief of interaction for pairs of proteins [5]. Although STRING can annotate the user’s novel genomes, it accomplishes this task using traditional annotation processing and sequence similarity. We believe that topological annotation’s main disadvantage based on sequence similarity resides in the novelty of new genomes. We knew that at least 10% of predicted genes from a recently elucidated genome are not present in previously annotated genomes [6]. This property implies that in a newly characterized *Escherichia coli* lineage, at least five hundred genes will not receive a single annotation if topological annotations based on sequence similarity (TABSS) are utilized. Therefore, we will miss approximately 2.5 million possible annotation interactions, or 10% of possible interactions, because these five hundred novel genes have no history. Producing a de novo annotation of the topological network for all new genomes assembled is not a practical solution. Instead, we need to gather a representative set of genomes of each genus or species to produce high-confidence de novo topological annotations [7]. The main bottleneck to this approach is processing power. Even if processing power were not the main issue, the time for doing it would become the next pressing issue because we have several new genome lineages available daily. To overcome such bottlenecks, we propose a new bioinformatic tool, named GENPPI, that is capable of processing a set of genomes stored in a conventional configuration machine.

## Implementation

### Genomes studied

We obtained the genomes investigated in this work using the official NCBI file transport protocol. We listed the genomes by their GenBank, assembly code, and assembly version, for *Corynebacterium pseudotuberculosis* and *Corynebacterium diphtheriae*. We also included a nickname to enable easy identification across the results section; the nickname is surrounded by parentheses: GCA_001457455.1_NCTC11397 (Cdip), GCA_001833005.2_ASM183300v2 (Cdip01), GCA_002073375.2_ASM207337v2 (Cdip02), GCA_004758745.1_ASM475874v1 (Cdip03), GCA_900638705.1_59178_D01 (Cdip04), GCA_902497465.1_YE-NCPHL-90 (Cdip05), GCA_004771215.1_ASM477121v1 (Cdiplaus), GCA_000144935.3_ASM14493v3 (Cp1002B), GCA_000233735.1_ASM23373v1 (Cp106A), GCA_000265545.3_ASM26554v3 (Cp162), GCA_000144675.2_ASM14467v2 (Cp231), GCA_000263755.3_ASM26375v3 (Cp258), GCA_000258385.1_ASM25838v1 (Cp267), GCA_000248375.2_ASM24837v2 (Cp316), GCA_000259155.4_ASM25915v4 (Cp31), GCA_000241855.1_ASM24185v1 (Cp3995), GCA_000227175.1_ASM22717v1 (Cp4202A), GCA_000227605.3_ASM22760v3 (CpCIP5297), GCA_000143705.2_ASM14370v2 (CpFRC41), GCA_000152065.3_ASM15206v3 (CpI19), GCA_000255935.1_ASM25593v1 (CpP54B96), GCA_000221625.1_ASM22162v1 (CpPAT10), GCA_000730445.1_ASM73044v1 (CpString).

### Metrics and reference genomes

To test the validity of the results observed within the GENPPI interaction networks, we performed trials with variations in the following parameters. We describe metrics 1–5 as the following:

1. Number of nodes/vertices: number of proteins present in the network;
2. Average degree: number of existing interactions compared to the number of proteins;
3. Density: ratio between a total number of edges and possible edges according to the number of vertices;
4. Number of edges: number of interactions between the proteins in the network;
5. Maximum degree: number of interactions that the most interactive protein has within the network.

We obtained the interaction networks of a set of genomes from model organisms from the STRING database. We calculated these metrics using the software GEPHI and ordered the columns according the level of importance (Table 1). Among ours objectives for the interaction networks created by GENPPI is the study of centrality measures. We believe in the necessity of utilizing differences in degrees to create vertices with distinct metrics, thereby avoiding the same centrality values. Thus, we understand that encouraging interaction networks for studies of centrality measures requires a nonuniform distribution of probabilities regarding their vertices’ degrees. This principle dictated our way of thinking about the parameters that would classify talented interaction networks regarding the study of centrality measures. We define the set of metrics in this section as parameters of a network’s desired quality level. Metric 1 (M1) should represent as many genes as possible for further analyses. We do not want large numbers for M2, M3, and M4 in order to minimize the risk of low discrimination power for centrality measures. For the sample of genomes depicted in Table 1, most of each genome’s genes are accounted for, and we have modest values for metrics from M1 to M5.

**Table 1:** Sample of model organisms obtained from the STRING database according to seven network metrics

Organism	STRING nomenclature	Vertices	Average degree	Density	Edges	Maximum degree
*Escherichia coli*	ATCC 8739	4190	195.34	0.047	409,238	1697
*Bacillus subtilis*	subsp. subtillis	4181	244.388	0.058	51,0983	2014
*Caulobacter crescentus*	CB15	3721	208.14	0.056	387,245	1393
*Mycoplasma genitalium*	ATCC 33530	474	128.35	0.271	30,419	318
*Synechocystis (Cyanobacteria)*	sp. ATCC 27150	4124	215.33	0.052	444,011	1548
*Pseudomonas fluorescens*	NCIMB 11764	6384	247.91	0.039	791,330	2158
*Azotobacter vinelandii*	DJ	4955	233.558	0.047	578,640	2047
*Streptomyces coelicolor*	A3(2)	7741	357.142	0.046	1,382,317	3576

We define these networks as appropriate to infer centrality measures. The majority of the networks from this sample have densities less than 0.100. The average degree, density, and edges were 228, 0.077, and 567 thousand, respectively

**Table 2:** With values 1 and 25 for the aa-limit and check-limit parameters, respectively, our heuristic guarantees a minimum identity percentage equal to 92.55% for pairs of similar classified proteins (Table 3)

Amino acids	A	R	N	D	C	E	Q	G	H	I	L	K	M	F	P	S	T	W	Y	V
A histogram	12	2	8	6	4	10	1	9	2	11	6	13	3	2	4	14	5	1	5	10
B histogram	11	3	8	6	4	11	1	9	2	11	8	12	3	2	4	13	4	1	4	11
abs(A-B):	1	1	0	0	0	1	0	0	0	0	2	1	0	0	0	1	1	0	1	1

According to the heuristics of GENPPI, proteins A and B are similar because, in the difference of their amino acid histograms, at least 25 of the 26 possible types presented frequency differences less than or equal to 1. In this table, we present only the 20 principal amino acids for the sake of exemplification. For the proteins A and B, in fasta format below, we have 94.5% identity (96.9% similar) according to the Needleman–Wunsch Algorithm. Amino acids in bold format are the different ones between A and B sequences

>A Protein

MAYSKKVMDHYENPRNVGSFSNSD**N**NVGSGLVGAPACGDVMKLQIKVNE**K**GIIEDACFKTYGCGS

AIASSSLVTEWVKGKSI**T**EAESIRNTTIVEELELPPVKIHCSILAEDAIKAAI**A**DYK**S**KK**YS**N

>B Protein

MAYSKKVMDHYENPRNVGSFSNSD**L**NVGSGLVGAPACGDVMKLQIKVNE**E**GIIEDACFKTYGCGS

AIASSSLVTEWVKGKSI**V**EAESIRNTTIVEELELPPVKIHCSILAEDAIKAAI**S**DYK**R**KK**NL**N

### Novel heuristic for faster sequence proteins comparing

In our software GENPPI, we represent the proteins through an amino acid histogram, which indicates the amino acid frequency distribution within a protein sequence (Table 2). In the process of comparing two proteins, we applied our similarity heuristic approach, known as Histofasta checking (Algorithm 1). We based HistoFasta on an amino acid frequency difference. Therefore, to match the similarity between proteins, our heuristic uses two parameters. The first is the aa limit (*-aadifflimit*), meaning the tolerated histogram difference for an amino acid frequency. The second one is check-limit (*-aacheckminlimit*), representing the maximum number of amino acids that can acceptably have the *-aadifflimit*. For instance, considering hypothetical proteins A and B, we first created an amino acid histogram for these two proteins (Table 2). Next, we checked the similarity between A and B sequences, comparing the difference between their amino acid histograms and verifying the number of different amino acid frequencies within the tolerated limit. A pair of similar proteins needs to guarantee minimal identity. To achieve minimal identity through HistoFasta checking, we performed exhaustive comparisons to the Needleman–Wunsch algorithm [8]. For this comparison, we used the *Mycobacterium tuberculosis H37Rv* lineage, simulating the search for homologous proteins within this genome. We varied our heuristic parameters to find configurations generating satisfactory minimal percentages of identity to the Needleman–Wunsch algorithm. Table 3 shows the top values for our heuristic parameters generating high percentages of minimum identity for the pairs of proteins classified as similar. For illustration, if out of the 26 possible amino acids, there are at least 25 (*-aacheckminlimit 25*) whose frequency difference is at most one (*-aadifflimit 1*), such a pair of proteins are classified as similar with at least 92.55% of amino acid identity. With HistoFasta checking, GENPPI can verify the relevant similarity of proteins and construct worth pangenomes. HistoFasta checking consistently achieves the constant time complexity of *O*(26). At the same time, the Needleman–Wunsch algorithm has a complexity of *O*(*nm*), where the aligned sequences have sizes of *n* and *m* letters. 
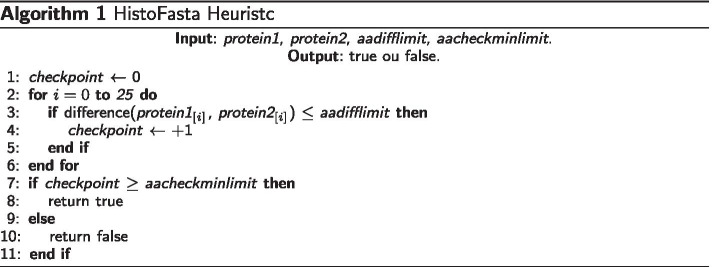

**Table 3:** Comparison of our heuristic to find high similarity pairs of proteins (HistoFasta) to the exact algorithm Needleman–Wunsch

AA limit (-aadifflimit)	Check limit (-aacheckminlimit)	Number of similar proteins	Mean identity	Median identity	Min identity
0	26	336	100.00	100.00	100.00
0	25	336	100.00	100.00	100.00
0	24	360	99.95	100.00	97.96
0	23	366	99.91	100.00	96.94
0	22	368	99.90	100.00	96.94
0	21	370	99.87	100.00	94.68
0	20	372	99.83	100.00	91.75
0	19	382	99.60	100.00	85.57
1	26	360	99.95	100.00	97.87
1	25	370	99.84	100.00	92.55
1	24	390	99.07	100.00	29.21
1	23	428	96.21	100.00	29.21
1	22	500	89.38	100.00	17.33
1	21	784	71.68	97.70	17.33
1	20	2164	52.27	39.60	17.33
1	19	6120	43.26	36.36	15.00

For the creation of the core pangenome, we need only the higher matches

**Table 4:** GENPPI execution line with parameters to generate interaction networks keeps fixed expansions for conserved neighbourhoods and variations controlling the number of phylogenetic profile interactions

Id	Parameters
f1	genppi -expt fixed -w1 10 -cw1 3 -ppiterlimit 1000000 -ppdifftolerated 3 -ppaadifflimit 0
f2	genppi -expt fixed -w1 10 -cw1 4 -trim 20000
f3	genppi -expt fixed -w1 10 -cw1 1 -ppiterlimit 500000
f4	genppi -expt fixed -w1 10 -cw1 1 -ppcomplete -aadifflimit 0 -aachecklimit 24
d5	genppi -expt dynamic -ws 3 -ppcomplete -ppdifftolerated 1 -pphistofilter

We omitted the folder parameter (-dir) for not contributing changes in the nodes or edges’ volume resulting in the networks. Execution d5 is a dynamic expansion for the conserved neighbourhood. d5 has no counterpart results of a fixed retraction. d5 was maintained in this table solely to group the documentation on the exploited commands

### Complexity analysis of the Dynamic Expansion for Conserved Neighbourhood algorithm

The Dynamic Expansion for Conserved Neighbourhood (DECN) algorithm (Algorithm 2) inspects genomes in a forward sense according to the disposition of protein sequences in a multifasta file. It works simulating the traversing of pairs of DNA strands, using a pivotal genome as a reference, sequentially reading open reading frames, and looking for neighborhood conservation. As a prerequisite, we must adequately order protein sequences in multifasta files, just like in their respective origin nucleotide sequences. The DECN algorithm consists of four nested repeating commands, one of which does not have a specific variable whose limit stipulates the end of repeating execution. We calculate the complexity of GENPPI through the DECN algorithm. The first loop in Algorithm 2 (line 1) ensures that DECN will inspect all proteins of the pangenome. The number of proteins for a set of genomes depends on the average number of proteins per genome ($\nu$) multiplied by the number of genomes ($\mu$). In line 2, one protein becomes the pivotal one for CN analyses. In line 3 we define the dynamic list gene-conservation. It keeps a list of how many times the algorithm found each gene as conserved, starting from the pivotal one in the current neighborhood under analysis for all genomes. We update the gene-conservation list each time the algorithm finds a conserved gene in one of the genomes under inspection. The ws variable determines the initial size of the gene-conservation list, which can get bigger. The value of 1 means that there is conservation in the windows of ws size at least within the pivotal genome. As HistoFasta heuristics (Algorithm 1) work on protein pairs, in line 4, the second DECN loop selects a homolog protein (pivot-2) to pivot-1 to check for a conserved neighborhood in another genome. As the algorithm inspects other genomes via pivot-2 (line 4), the gene-conservation list can increase the occurrence of genes in the current window. In line 4, the estimated size of the vector of proteins similar to the pivot depends on $\mu$ multiplied by the mean similarity between genomes at the protein level ($\sigma$). The GENPPI -ws parameter, set at run time, defines the value of the variable ws (window size of CN analyses) at line 8, specifying the execution limit of the fourth inner loop. Whenever HistoFasta (line 9) estimates a similar protein pair among genomes, the algorithm should increment the level of conservation (line 11) in a neighborhood and expand the boundaries of the neighborhood for the subsequent interaction loop analysis (line 12). 
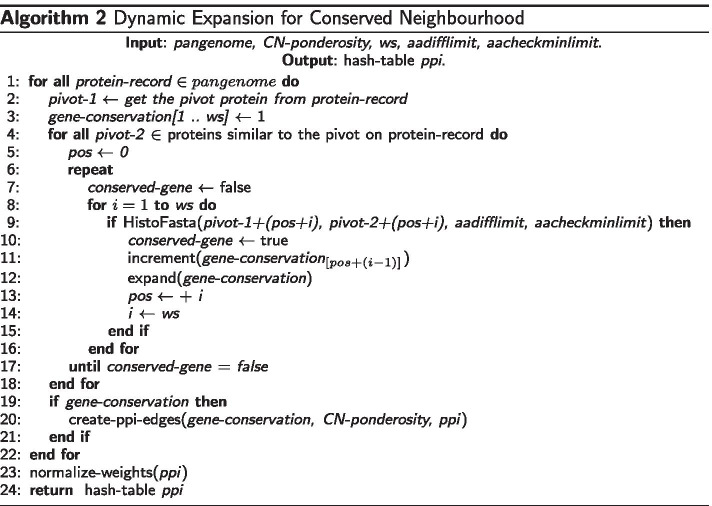


In Algorithm 2, a ws value means a conserved neighborhood defined with this initial conservation window limit. Suppose DECN finds a minimal number of proteins conserved within an initial window of ws size. In that case, it registers the last conservation achievement and prepares to check for an incremented value of ws for the next set of ws proteins in the vector proteins similar to the pivot. We do not need to reanalyze the previous proteins with the initial ws value. We achieve this forward walkthrough by monotonically incrementing the variable *pos*. To better explain the lines 11 and 12 in the DECN algorithm, lets suppose the ws parameter equal 3. In this case, gene-conservation is initiated with the value of (1 1 1) in line 3. If in the first iteration DECN finds that the subsequent neighboring gene of pivot-1 is conserved in another genome, the vector is incremented in that position and expanded, thus becoming: (2 1 1 1). Whenever DECN finds gene conservation, it first increments the vector value in that position (line 11), expands it with the amount of 1’s required for the next iteration (line 12), and resumes the expansion of the following gene onwards. The criterion of expansion stop is to check ws subsequent genes without verifying any preserved gene. If in step 2 of the expansion conservation is found, DECN increments the vector in that position and expands it by inserting two more equal values 1, thus being: (2 2 1 1 1). In the next iteration, DECN continues to expand the following gene (step 3) onwards. Thus, if in the last expansion step (5) another conservation is found, the algorithm increases the vector in that position and inserts three more values equal to 1, thus: (2 2 1 1 2 1 1 1). Whenever DECN finds conservation in an expansion step, it increments the list in that position and prepares it by inserting the required amount of 1’s for the next iteration.

In line 6, there is a loop whose terminating condition (line 17) is responsible for the uncertainty about the DECN time complexity. The termination of DECN for a pair of genomes will happen when a current stretched window no longer gets a minimal percentage of conserved proteins within it. We cannot infer an exact formula regarding the number of interactions starting at line 6. However, we could try to tabulate it incrementing the ws value for a set of genomes. We named this variable $\rho$. After all, the two innermost loops of Algorithm 2 have a complexity proportional to $\rho$ multiplied by *ws*. We list the variables that influence the algorithm for calculating CN in DECN.

- $\nu$ = average number of proteins among the genomes analyzed;
- $\mu$ = number of genomes analyzed;
- $\sigma$ = mean similarity between genomes at the protein level;
- *ws* = window size and step for dynamic expansion in DECN algorithm;
- $\rho$ = a constant specific for each set of genomes.

Given a pair of knowing $\rho$, $\nu$, and $\sigma$ values, we could try to approximate $\rho$ for a particular set of genomes. $\rho$ has values that are proportional to $\nu$ and $\sigma$. We could, for instance, set up a quadratic system of linear equations (1) that could allow approximating values of $\rho$. In Eq. 1, suppose the data of the knowing genomes are from *Corynebacterium* (Cp) and *Staphylococcus* (St).

1 $$\begin{aligned} \begin{aligned} {\left\{ \begin{array}{ll} K_\sigma \sigma _{Cp} + K_\nu \nu _{Cp} = K_{Cp} \\ K_\sigma \sigma _{St} + K_\nu \nu _{St} = K_{St} \end{array}\right. } \end{aligned} \end{aligned}$$

Once estimated the constants of the Eq. (1), It could be possible in estimating the value of $\rho$ ($\nu$, $\sigma$) with the Eq. 2 for a genome (g):

2 $$\begin{aligned} \rho _g ( \nu _g, \sigma _g)= K_\nu \nu _g + K_\sigma \sigma _g \end{aligned}$$

The DECN complexity was infered by the presence of the below relations per line at the Algorithm 2:

- $$\nu \mu$$ : line 1
- $$\mu \sigma$$ : line 4
- $$\rho ws$$ : lines 6 and 8

Finally, the amount of comparations made between the proteins of a set of genomes for the neighborhood algorithm conserved with the dynamic expansion (DECN) is estimated with the Eq. 3.

3 $$\begin{aligned} O ( \nu \mu ^{2} \sigma (\rho (\nu , \sigma )) ws ) \end{aligned}$$

We emphasize that these are estimates. For example, for *Staphylococcus*, with *ws* ranging from 5 to 7, we spent 68, 96, and 102 h, respectively. There is no guarantee that there will be a monotonic increase in the complexity of one value from *ws* to another higher, that is, that we can count on the repetition factor $\rho$ ($\nu$, $\sigma$) will always be maintained. There is no constant difference between executions from *ws*=5 to *ws*=6 (28 hours) and from *ws*=6 to *ws*=7 (6 h). It characterizes an uncertainty in the number of runs of our algorithm. However, we can count on an average value for this inflation of executions between different values of *ws*.

### Complexity analysis of the conserved phylogenetic profile algorithm

Considering that Histofasta, DECN, and Conserved Phylogenetic Profile are the principal algorithms of this work, we here documented the conserved phylogenetic profile (CPP) algorithm procedure. The CPP algorithm inspects the genomes looking for genes co-occurring in several genomes, despite their physical dispositions.

In line 1 of Algorithm 3, we initialize a phylogenetic-profiles hash table, a list of profiles’ proteins. We use this hash table to store the phylogenetic profiles of conserved proteins in genomes. The generate-profiles function of line 2 scans the genomes table-hash by assembling proteins’ phylogenetic profile. CPP identifies similar proteins by applying the HistoFasta heuristic, called by the function generate-profiles, for all possible protein pairs. Using the variables defined in the DECN algorithm, HistoFasta performs $(1/2)(\nu (\nu -1))$ comparisons. The generate-profiles function considers all genomes included in the analysis at once. Assuming that we included six genomes in one analysis, the phylogenetic profile of a protein in a query genome would be a 5-bit chain, each representing the presence (1) or absence (0) of a similar protein in one of the five subject genomes. At the end, line 2 demands $(1/2)(\nu (\nu -1))(\mu -1)$ comparisons. In the loop in line 3, CPP will inspect the generated profiles for $\mu$ genomes. In line 4, CPP will group proteins with identical or similar (ppdifftolerated > 0) phylogenetic profiles at the cost of $\nu ^{2}$ comparisons. Line 5 will iterate through the groups of phylogenetic profiles. The iteration turns in line 5 will depend on the number of groups created for each genome. Here we should introduce a variable similar to the $\sigma$ used in the DECN algorithm since the number and the size of the groups depends on the evolutionary relationships of genomes under analysis but a $\sigma$ for phylogenetic profile conservation. In line 6, CPP will create protein interaction edges for all pairs of proteins. CPP ends after creating PPI edges for all possible pairs of proteins with the cost of $(1/2)\nu (\nu -1)$. However, we expect the number of comparisons in line 4 and 6 to be smaller than $\nu$ since we know not all proteins within a genome is conserved among several genomes. The $\nu$ can be considered the worst case, for instance, when comparing clonal genomes. A GENPPI user can easily perceive this smaller than $\nu$ behavior when running the program. In general, no matter the PP parameters, the program does not take too much time to finish the PP analyses but the Algorithm 2. Finally, the big O complexity for the CPP algorithm is estimated with the Eq. 4.

4 $$\begin{aligned} O ( \mu \nu ^{4} \sigma ) \end{aligned}$$

In line 4 of Algorithm 3, the agroup function has the ppdifftolerated parameter. This parameter determines whether clusters will be formed only of proteins with identical phylogenetic profiles (ppdifftolerated 0) or proteins with similar profiles (ppdifftolerated > 0). CPP considers two phylogenetic profiles similar if the difference of their bit chains is not more significant than the number of different bits tolerated by the ppdifftolerated parameter. For instance, If the tolerated difference is equal to 1, proteins whose phylogenetic profiles differ by a maximum of 1 bit will be considered proteins with similar profiles. By default, GENPPI predictions by the conserved phylogenetic profile method are made only for pairs of proteins with identical phylogenetic profiles. If necessary, a user should pass a non-zero ppdifftolerated parameter at execution time.
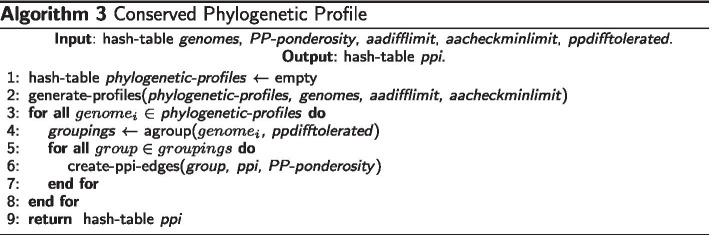


### Trustable results’ measure

**Fig. 1:**
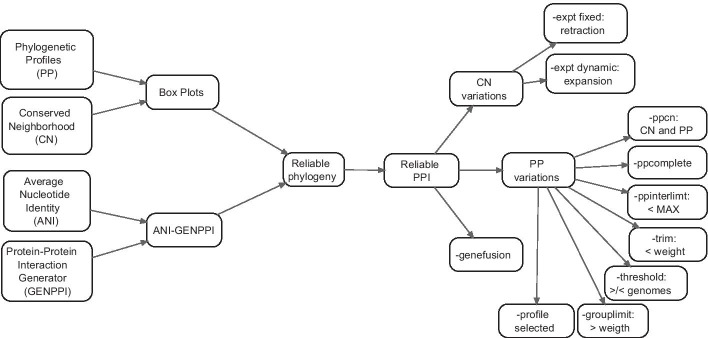
Scheme of an arrangement of the results. Suggesting that GENPPI produces trustable results

Figure 1 presents a scheme in which we attempt to explain the disposition of the results that we obtained with GENPPI. Since the GENPPI program can show neighbourhood conservation or phylogenetic profiles, the first step is to produce a pangenome. The data on this pangenome are not in the session of results but rather are results derived from the pangenome. In possession of a pangenome, GENPPI conducts a systematic search for neighbourhoods and conserved phylogenetic profiles. To direct this search, we start from proteins with a high identity (greater than 90%) or proteins with a high chance of belonging to a central or accessory genome under analysis. The point of this approach is to show that the central pangenome’s characteristics, phylogenetic profile (PP), and conserved neighbourhood (CN) are trustable; otherwise, they would not correctly represent facts about the evolutionary relationship of known bacterial species. Once the correction of evolutionary relationships is confirmed, we can explore distinct ways of generating these networks. In brief, the network creation process variations stem from limitations that we can attribute to how many interactions we want to be part of formatted networks to answer a specific scientific question. However, regardless of the level of data restriction imposed by the user to answer their scientific query, we ensure that the networks produced by GENPPI are trustable because they represent, with a high degree of confidence, the evolutionary relationships of the bacterial species under analysis.

### Parameters for interaction networks created with GENPPI for *Corynebacterium*

We try to provide complete control to the user via parameter passing to GENPPI. We implemented 29 possible parameters to our software. The number of possibilities one could exploit is far beyond this article’s purpose. However, we empirically made such a search through the space search of our commands. We listed the final result of our quest in Table 4. One should keep in mind the three primary parameter sets: Conserved Neighborhood (CN), Phylogenetic Profile (PP), and the last for Gene Fusion (GF). In this work, we did not focus on GF since, for our case study, it simply doubles our software’s computation and adds few dozen interactions to the final set of PP and CN. One should keep in mind that interactions created by these three significant sets are independent: no PP parameter will interfere with the CN results and vice-versa. The consequence is a whole different set of parameters for CN, but keeping the same PP parameters always will provide the same output concerning PP. Another result is that a pair of proteins could have three different interactions, one for each primary set of parameters. We did not implement a mechanism to join the group of interactions for a pair of proteins. Table 4 lists the GENPPI execution commands that produced the networks listed in Table 5. All settings were derived from an initially fixed window (CN) of *-w1 10*, meaning that ten proteins in a window were analysed sequentially for their conservation in all genomes under analysis. For example, -cw 4 indicates that four proteins were conserved in a neighbourhood, and all proteins were considered to be related to interaction. However, if we could not find conservation in an analysed window, making use of the *-w1* and *-cw1* parameters, then GENPPI reduced the size of the *-w1* window to *-w2* and the minimum quantity *-cw1* for *-cw2* parameters, as automatically configured by the program, and the patterns was repeated until the smallest window possible was explored. For this reason, when we refer to fixed expansion parameters, GENPPI performs a retraction to smaller window values and a smaller minimal threshold of acceptable similarities to annotate neighbourhood conservation. An interaction weight is associated with proteins said to be interacting, and it is proportional to the distance between proteins within a window. When we use the *-cw1 1* parameter (CN), we consider windows containing any conserved proteins. In Table 5, the association of high window size (*-w1*) with a low number of required conserved genes (*-cw1*) generated adequate node numbers in the four best runs compared to STRING. The set of parameters of f1 id was responsible for the highest value in all metrics. Concerning phylogenetic profile, we noted that changes in the maximum number of desired interactions (*-ppiterlimit*) from one to half million in the f3 id reduced all the metrics’ values from 1 to 7, including the density to visualize a network nuance in topology. The parameter for the absence of filters for phylogenetic profiles (*-ppcomplete*) was relevant in the execution of the f4 id. The number of nodes was close to that found by STRING. Still concerning PP, only using a limit parameter of the maximum number of interactions (*-trim*) enabled an empirically sought density value of less than 0.1 to be obtained. The *-trim 20000* parameters in the f2 id enabled a density value of 0.034, a number lower than that found by the STRING reference network. It is also interesting to note that the set of parameters of the f4 id provided atractive values for all metrics, except for the maximum degree, which was nearly half that of other results.

## Results

### Heat-maps

**Fig. 2:**
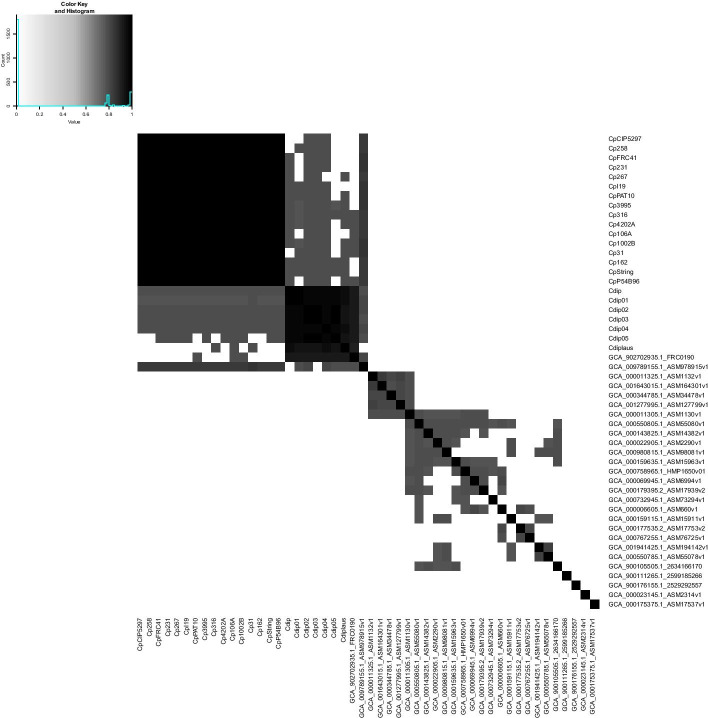
Average nucleotide identity for 50 genomes of the genus *Corynebacterium*. The largest grayish square represents the clusters of *C. pseudotuberculosis* and *Corynebacterium* *diphtheriae*. Both groupings have units that are almost black due to the high score of DNA similarities

**Fig. 3:**
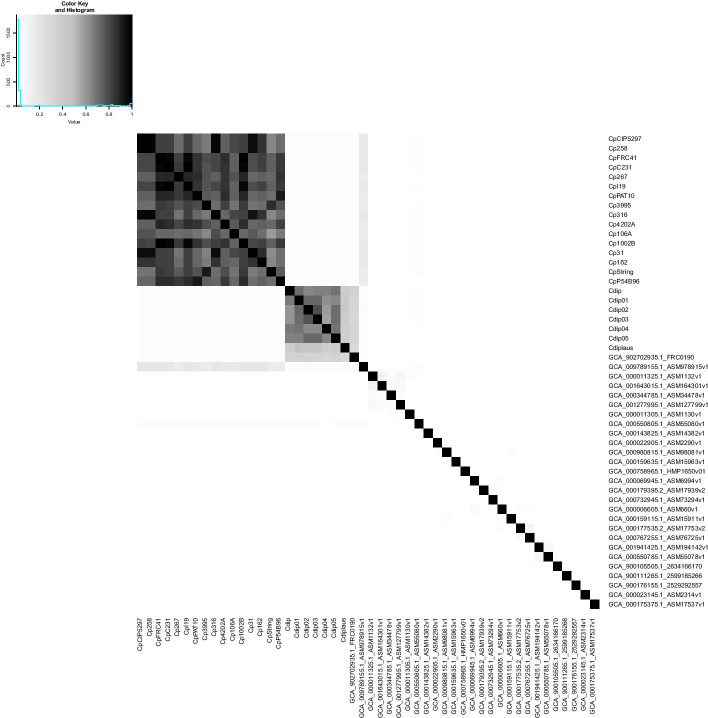
Pangenome similarity profile for the same 50 genomes of the genus *Corynebacterium* depicted in Fig. 2. The clusters of *C. pseudotuberculosis* and *Corynebacterium* *diphtheriae* are the most grayish. The remaining units are whitish due to the low protein similarities of their phylogenetic profiles

**Fig. 4:**
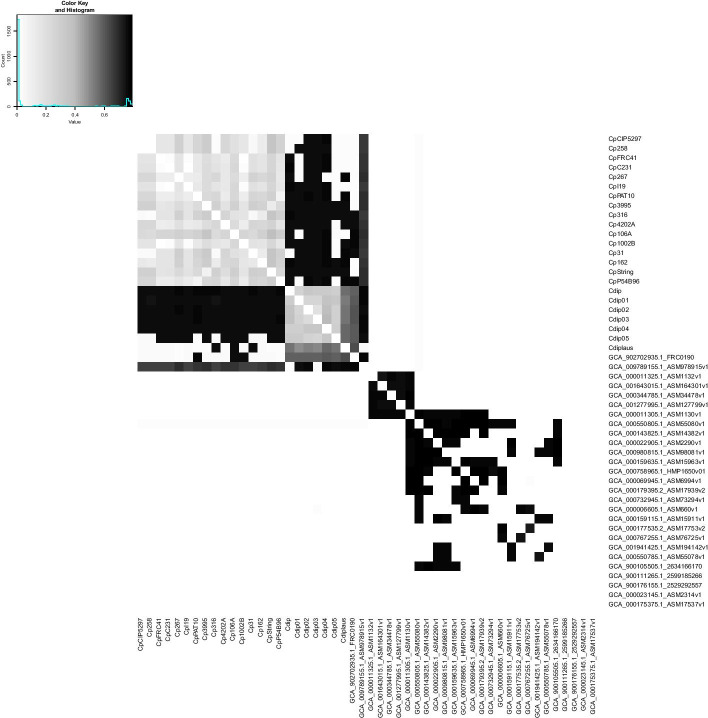
Profile differences between Figs. 2 and 3. It accounts for the chunk of divergence about ANI and the pangenome raised by GENPPI. Black cells represent the maximum difference, while white cells account for smaller differences, with grayish units representing intermediate differences. The majority of the comparisons are white because ANI and GENPPI agree on the low similarity of the compared genomes and small differences

The analysis of the difference between genomes using nucleotide sequences, known as Average Nucleotide Identity (ANI), is presented in Fig. 2. Figure 3 depict the results of GENPPI for the same genomes. However, the data used in Fig. 3 show the extent of proteins shared between each pair of genomes. For example, suppose genome A has 2200 proteins. Of this total, 2000 proteins of genome A have high similarity to proteins of genome B. Therefore, at row A and column B of the heat graph, we have 2000/2200 = 0.91 % protein similarity between genomes A and B. Note that in row B and column A, the protein similarity value between these genomes is likely to be specific. We explain this difference as occurring because the denominator is the measure of B proteins, and the numerator is the chunk of B proteins found in A. The cell colours above and below the main diagonal depend on which genome is the numerator and which is denominator. In Figs. 2 and 3, we chose the colours white and black for low and high identical genomes, respectively. The gray colour is an intermediate value between white and black. Genomes of correlated species compared by ANI are differentiated by small percentages and are generally above 90% (Fig. 2). Values of protein similarity between the pangenome (Fig. 3) were less sharpened than the ANI values. A rate of less than 50% can be a high similarity value between a pair of genomes. The majority (87%) of the possible combinations obtained from the 50 genomes of the genus *Corynebacterium* have a similarity of less than 50% (data not showed). Figure 4 represents the differences between the similarities of each pair of genomes, as determined by ANI (Fig. 2)—GENPPI (Fig. 3). Importantly, the differences indicated in Fig. 4 are not regarding the similarity between the species but how much GENPPI and ANI on these species agree or diverge. In Fig. 4, heat map cells with black values indicate a very pronounced difference, while white values indicate a slightly significant difference between ANI and GENPPI. Most of the units that constitute the *C. pseudotuberculosis* grouping are white. Other units are slightly grayish, representing differences with little expressiveness, between ANI and GENPPI. Excluding the Cdiplaus genome, the *Corynebacterium* *diphtheriae* cluster would have a colour pattern similar to that of *C. pseudotuberculosis*. Some cases are noteworthy in Figs. 2, 3 and 4. (i) The genome identified as GCA_902702935.1_FRC0190 refers to *Corynebacterium rouxii* (high GC Gram+). This genome showed high similarity at both the nucleotide and protein levels with the *C. diphtheriae* grouping. An analysis of the data from the heat maps of our work indicates that the genome named *C. rouxii* was *C. diphtheriae*. In addition to our analyses, the specialized literature in these organisms confirms our recommendation to change the nomenclature from the species *C. rouxii* to *C. diphtheriae* (Badell et al., 2020). (ii) The genome identified as GCA_009789155.1_ASM978915v1 refers to *Corynebacterium ulcerans*, strain MRi49. According to the ANI analysis, this genome exhibited high similarity at the nucleotide level with the clusters of *C. pseudotuberculosis* and *C. diphtheriae*. However, the genome exhibited a higher similarity at the protein level with *C. pseudotuberculosis*. Nevertheless, given that we can perceive a slight gray colour in the GENPPI heat map, we believe that this species has some protein similarity to *C. pseudotuberculosis*. In this case, the literature describes the species *C. pseudotuberculosis*, *C. diphtheriae*, and *C. ulcerans* as being evolutionarily related (Busch et al., 2019; McNamara, Cuevas, and Songer 1995). Most of Fig. 4 is coloured white, meaning that the ANI enables us to reach the same conclusion as GENPPI regarding the minor similarity between the majority of the possible relationships between each pair of genomes. However, there is a considerable portion of Fig. 4 that is in black colouration. The colour reflects similarities found at the nucleotide level that do not sustain themselves at the amino acid level compared with the pangenome analyses of GENPPI. It is interesting to note that for the clusters of *C. pseudotuberculosis* and *C. diphtheriae*, the pattern of similarity between ANI and GENPPI is notable, despite the presence of other numerical values. By guarding the differences in the similarity quantities, we reach the same conclusions between Figs. 2 and 3 regarding the evolutionary proximity of cluster organisms. Using the ANI results (Fig. 2), we can note similarities between genome sequences not reflected in the pangenome (Fig. 3). Such closeness extends beyond the clusters of *C. diphtheriae* and *C. pseudotuberculosis*. Therefore, our results support the hypothesis that the similarity between species using the protein pangenome is more useful for differentiating them compared to the DNA sequences. This finding is reasonable because we have demonstrated in Fig. 3 that the species are distinctive with distinct pangenomes, despite having similar DNA sequences, as depicted in Fig. 2. The differentiation of proteins is known to occur because of transcription in the DNA strands. Therefore, a phylogenetic analysis using the pangenome helps to more accurately determine differences between species compared with an identical study examining DNA. Nevertheless, when we analysed genomes from the same species, there was parity between phylogenetic analyses using ANI and GENPPI.

### Graph of boxes of conserved phylogenetic profiles

**Fig. 5:**
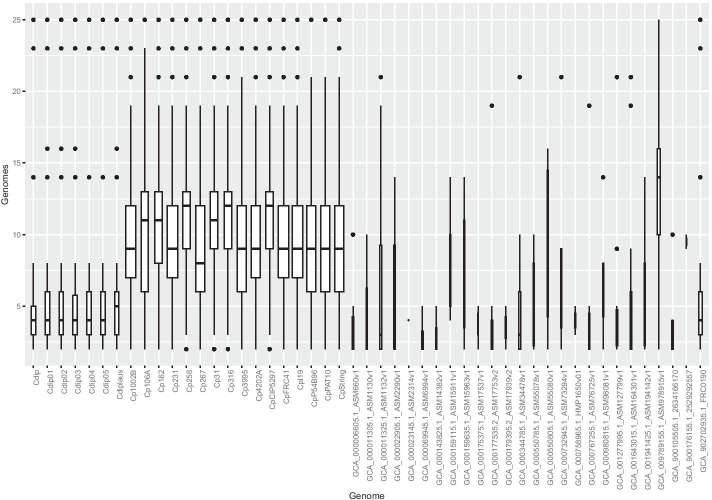
Each genome has a box plot registering stats for their conserved phylogenetic profiles. The width of a box plot is proportional to the number of PPs found. There is a numerical expressiveness of genomes from *C. pseudotuberculosis* (left) and *diphtheriae* (leftmost). For the former, the PP median enables separation of the biovars ovis and equi (highest medians)

Figure 5 summarizes the phylogenetic profiles present in each genome analysed (Genome) versus the bulk of genomes in which these profiles appear (Genomes). Therefore, the Y-axis is on the scale from 0 to N, where N is the total genome. In this graph, a median means a load of genomes in which we found conserved phylogenetic profiles, and the width of a plot box is proportional to the number of profiles conserved in a genome. The analysis of conserved phylogenetic profiles made by GENPPI demonstrated the relationship between the ovis and equi biovars of *C. pseudotuberculosis*. The biovar equilinage has six genomes: Cp106A, Cp162, Cp258, Cp31, Cp316, and CpCIP5297. We employed the median and first and third quartiles of genome box plots to demonstrate biovar equi separation. The equi biovar is represented by the first quartile of the plot boxes of the genomes aligning near the median of the plot boxes of genomes belonging to the biovar ovis. The genomes of the biovar equi that fall into this scenario are the following: GCA_000265545.3_ASM26554v3 (Cp162, from a camel in Egypt), GCA_000263755.3_ASM26375v3 (Cp258, from a horse), GCA_000259155.4_ASM25915v4 (CpCp 31, from a buffalo), GCA_000248375.2_ASM24837v2 (Cp316, from a horse in the USA) and GCA_000227605.3_ASM22760v3 (CpCIP5297, from a horse in Kenya). The exception to this rule was the genome with end GCA_000233735.1_ASM23373v1 (Cp106A, from a horse in the USA), which presented the first quartile closest to the ovis biovar strains. However, the median Cp106A was observed to be closer to the biovar equi. The expanded box plots are sixteen and comprise the species *C. pseudotuberculosis*. The box plots of the genomes of the species *C. diphtheriae* are seven and have a smaller width than that of *C. pseudotuberculosis*. Even because these box plots are less represented in this set of genomes, the other species did not show expressive phylogenetic conservation, and we presented plot boxes with a small width. These other species have only one genome representing them in this set of 50 from the genus *Corynebacterium*. Genomes numerically underrepresented compared to *C. pseudotuberculosis* and *C. diphtheriae* account for phylogenetic profiles preserved solely for the genus *Corynebacterium*. The *C. diphtheriae* and *C. pseudotuberculosis* clusters, on the other hand, dominate the number of conserved phylogenetic profiles. We utilized the species *C. diphtheriae* as a reference genome to assemble the first fifteen genomes of *C. pseudotuberculosis*. At the time, we believed that the species *C. diphtheriae* and *C. pseudotuberculosis* were very similar. At the end of the first assembly, we concluded that these species had a similarity level above 60% at the protein level. For the first automatic annotation transfer, this level of similarity was satisfactory. However, in Fig. 3, the colouration of protein similarity between Cp1002 and Cdip can be observed to be intense white staining, which reflects 2.4% protein similarity with a confidence level greater than 90% of the pangenome. This similarity is low because we set the program to raise the pangenome between these two strains to consider proteins similar only if they had more than 90% identity at the amino acid level. If we had decreased the criterion for determining resemblance, there would probably be a greater affinity between these two species. However, if we had diminished the stringency for proteins’ identity to nearby levels, 60% GENPPI would not translate such a set, given the pangenome’s reliability. With low levels of similarity, preserved protein domains that are present in many proteins with distinct functions could lead to false positive results regarding the pangenome’s central genome. The previous section showed the utility of generating a central genome with the ability to create phylogenetic clusters consistent with our biological knowledge of bacterial species. The analysis of the box chart results in Fig. 5 shows that phylogenetic profiles made by GENPPI are also consistent with the previous findings regarding species and biovars. Thus, the interaction networks created by GENPPI using the conservation of phylogenetic profiles can help us to identify a topological structure with biological significance.

### Box plot of preserved gene neighbourhoods

**Fig. 6:**
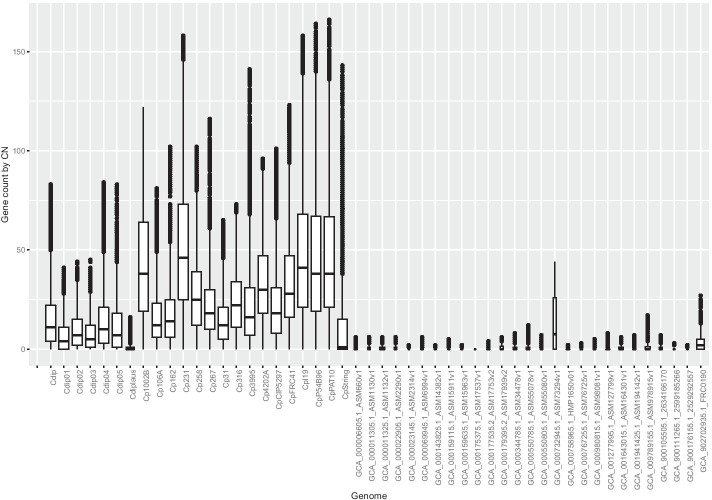
Better possible separation of the genus *Corynebacterium* achieved using the conserved neighbourhood. We achieved splitting via the expansion of a window of pitch three. The stopping criterion was a reduction by more than half in the number of conserved loci for window size. In this query, we did not ensure a full split of *C. pseudotuberculosis* biovars ovis and equi but of the main represented species

GENPPI does not work with the genomic DNA sequence but with a report exported from the DNA encoding proteins. However, the conservation of a gene’s DNA sequence location influences the box plot of preserved gene neighbourhoods. We assume that protein sequences tend to enter a multifasta file in an order similar to that observed when they were when extracted from a DNA sequence. GENPPI software receives as input a multifasta file of proteins ordered similar to the corresponding genes arranged on the DNA sequence. Given this premise, in Fig. 6, we use a window of size *w* to count how many genes are conserved according to at least some other *N* genomes under analysis. We store a conservation pattern if that pattern occurs in two or more genomes. Two very similar genomes may have almost identical gene neighbourhoods. For an example of two genomes evolutionarily close and assuming a value of *w* < 10, the median of a conserved neighbourhood (CN), the first quartile and the third quartile, as well as the maximum number of conserved genes, are all equal to w, except for several outliers. The greater the extent of a box plot is, the greater the number of genes with CN characteristics in a genome is. In a CN graph, there is no way to know which genomes are very similar. It is possible to know that there are very similar genomes with a minimum of two. When the GENPPI program runs without the restriction of the threshold window for conserved neighbourhood analysis with progressive increases of $-ws$ until the conservation quality decreases, we call this process a dynamic expansion. In Fig. 6, the measure of genes conserved in a neighbourhood (dynamic extension with $-ws$ 3) showed a high similarity between the genomes of the biovar equi of the species *C. pseudotuberculosis*, strains Cp106A, Cp162, Cp258, Cp31, Cp316, and CpCIP5297. The median of the six equine genomes remained below 25 genes. Within this graph, three out of sixteen genomes of *C. pseudotuberculosis* have box plots with the median below 25 not belonging to the biovar equi, the genomes Cp267, Cp3995, and CpString. We know the genomic relations between the biovars ovis and equi from the literature of *C. pseudotuberculosis* (Soares et al., 2013). When the dynamic expansion step $-ws$ is equal to 1, we have seven out of ten genomes of *C. pseudotuberculosis* biovar ovis whose medians approach those of the biovar equi genomes (data not displayed). However, if we increase the neighbourhood conservation window’s pitch, for example, to $-ws$ 5 and $-ws$ 7, there will be no changes against the result with $-ws$ 3 (data not displayed). Thus, the value that best created the separation of the biovars ovis and equi regarding the gene neighbourhood’s conservation was a dynamic extension step with window size equal to 3, value derived from experimentation and comparison between results. Nevertheless, in Fig. 6, when we utilized dynamic expansion, the seven genomes of *C. diphtheriae* had medians lower than the lowest median obtained for most *C. pseudotuberculosis* strains. The median of the *C. diphtheriae* species remained lower than the average of most of the species *C. pseudotuberculosis*. The Cdiplaus genome was at a median well below those of the other analysed genomes of *C. diphtheriae*. Considering that the literature reports Cdiplaus as a heterotypic synonym of *Corynebacterium belfantii* (Badell et al., 2020), we have evidence indicating that our analysis of the median genomes of *C. diphtheriae* would provide a correct classification of all genomes of the species *C. diphtheriae* analysed in this study. In addition, the difference between the CpString median compared to all other genomes of *C. pseudotuberculosis* and even with *C. diphtheriae* is noteworthy. In graphs of the number of genes per conserved neighbourhood generated by GENPPI, medians with values close to zero are found for genomes that have only one specimen per species among the analysed set. The GENPPI’s dynamic expansion to CN makes us pay the price for more accurate mappings. The number of protein comparisons is polynomial. The constant $\rho$ depends on the average number of proteins among the genomes analyzed. We spent 2 h finish considering a window size equal to three and 50 genomes. However, for *Staphylococcus*, with 57 genomes, we spent 32 h on the same window pitch. The counterpart of the dynamic expansion algorithm to CN is the fixed retraction. Instead of polynomial complexity, we have a logarithmic one, which takes about 40 min to process the identical 50 *Corynebacterium* genomes, considering an initial window of size 10.

### Comparing interaction networks created with GENPPI and STRING

**Fig. 7:**
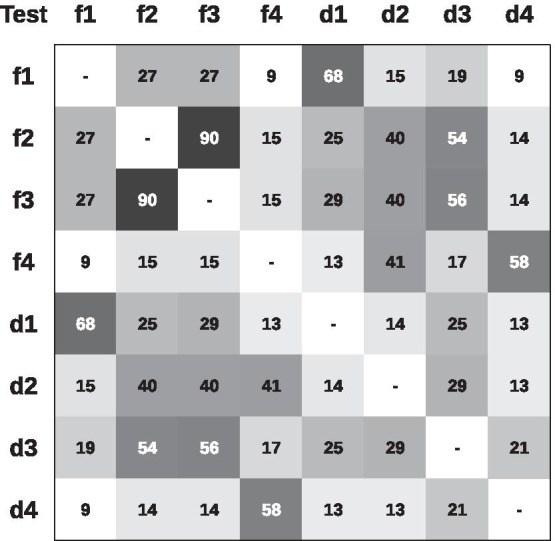
Quantity shared for each pair of runs of Table 5 regarding the first 100 proteins ordered by the metric Bridging Centrality. The fn and dn labels come from the executions of Table 5. White cells have a higher percentage of an intersection followed by yellow cells, and red cells have the lowest prevailing amounts between each gene neighbourhood parameter setting (fixed retraction or dynamic expansion)

We submitted a set of 50 genomes of *C. pseudotuberculosis* to several combinations of GENPPI parameters. The analyses were divided between the two types of window sets for a conserved neighbourhood (fixed retraction or dynamic expansion) versus the seven possible types of configurations for a boundary of phylogenetic profiles , including an option that does not restrict the load of interactions mapped in the final report. It is important to note that the code employed for assessing conserved PP (Algorithm 3) and CN (Algorithm 2) work independently. Each algorithm generates variant sets of interactions that can occur for the same pair of genes. We chose to explore CN execution variations without changing the PP execution mode. The objective was to facilitate the comparison between results. We assume the most relevant results produced by GENPPI were those with network metrics similar to STRING networks (Table 5). We believe that metrics like a more significant number of nodes and edges, plus lower density, medium degree, and maximum degree, are more suitable considering the centrality measures’ study. As an example of centrality measure favored by such a set of general metrics, we can cite Betweenness and Bridging Centrality, both dependent on the nodes’ degrees. For instance, imagining a highly connected network, we based our beliefs on the difficulty of making significant differentiation among the nodes. In this scenario, it would be arduous to pinpoint nodes with more topological significance in a network with a medium degree closer (for instance, a half or more) than the total number of nodes. We employed a network generated by STRING software for the genome of *C. pseudotuberculosis* as a reference for the metrics. Compared to the fixed expansion, the values of the metrics for dynamic expansion in Table 5 were approximate with point exceptions.The particular web created by d5 Id has a density and average degree above what we consider ideal for the study of centrality measures compared to the STRING reference. However, this network generated the best phylogenetic separation between species via CN (Fig. 6). This result is an example of the flexibility of network generation provided by GENPPI. Our software enables the creation of interaction networks customized for a user’s specific need, such as the study of measures of centrality (lower density) or the study of protein clusters (higher density). Regardless of the end-user objective and considering that interactions have a valid biological meaning, we guarantee the correction of the networks obtained in further studies. Given the variations in the bulk of vertices and edges that can compose each network created by GENPPI, we expect to experience diversity in the topology of nets created by our software. We present the results of an examination of topology’s variety in Fig. 7.

**Fig. 8:**
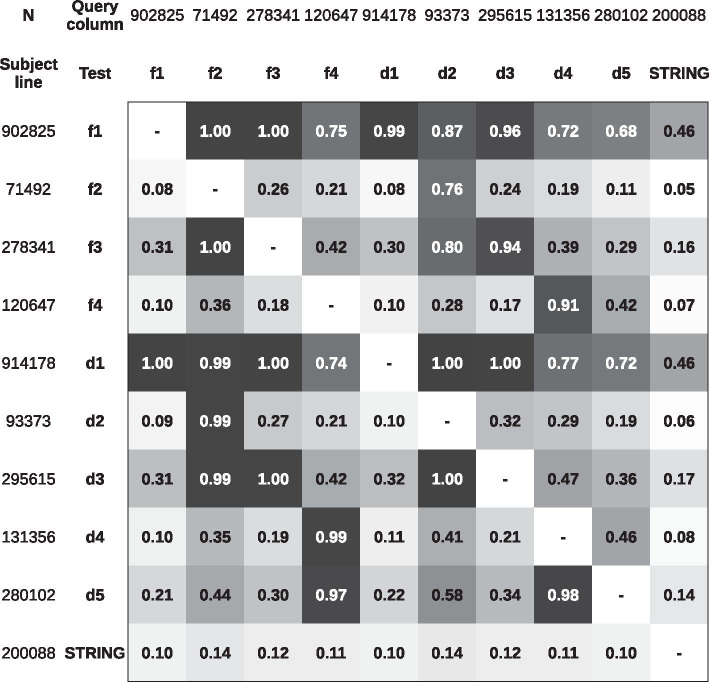
We query the network’s interactions listed in columns against subjects in rows. The result compares shared interactions between different GENPII parameters and the STRING. The more significant intersection of GENPPI obtained 46% of the STRING interactions (f1 and d1 Ids), even using 0.009% of genomes to craft the networks compared to the STRING database. On the other hand, the more significant result of the STRING network to our webs achieved 14%

In Fig. 8, we query the network’s interactions for each web in the columns against a subject in the rows. As a result, we compared the shared interactions between our products and the STRING output. The networks created by f1 and d1 Ids are the more numerous ones. The reason is that the parameters we set in these GENPPI’s executions allow exploiting a more significant number of possibilities. We justify such a conclusion because all other results we created, and even the STRING results, have the majority or a significant portion of their outcomes in the set of f1 and d1 outputs (dark grayish cells and values closer to one). We also accomplished the highest proximity of our networks to the STRING via f1 and d1 Ids. GENPPI identified almost half of all undirected edges mapped by STRING. On the other hand, the most significant number of edges STRING matched in the GENPPI’s results was 14%. Regarding the results in Fig. 8, the reader should note that we produced the GENPPI’s networks using solely fifty *Corynebacterium* genomes, a much smaller group of genomes than that used by STRING. On the other hand, the STRING database comprises five thousand and ninety genomes, including several other genera. It could explain the STRING intersections as the role of query or subject in Fig. 8. Considering we used less than 1% of the genomes hosted by the STRING site to generate our interaction networks, we claim as representativeness achieving almost half of STRING’s result. We also believe that if the STRING site uses our set of genomes, it could acquire a more remarkable intersection to our returns more significant than 14%.

### Topological diversity between *Corynebacterium* networks

There are no closed formulas for deciding on the quality of a network topology of a set of genomes. We used five criteria as guidelines (subsection Metrics and Reference Genomes) for selecting networks with an appropriate topology for biologically relevant analyses. For this reason, we focus on webs with metrics that are closer to the STRING reference. The following reasoning seeks to show that although networks use different topologies, our software networks can have topological similarities. This property is plausible, since the descriptors used are the same but appear numerically contrasting. Initially, we suspected that networks exploring the conservation of the gene neighbourhood by fixed retractions versus dynamic expansions would generate webs with topology sufficiently distinct that centrality metrics could lead one to question the quality of these networks from the biological perspective. We compared the top 100 proteins with the highest bridging centrality value of fixed and dynamic nets (Table 5). We present the results in Fig. 7 with a median and average of 25 and 29 intersections per pair of experiments, respectively. The mean and median values are overcome loosely by topologies created from combinations of fixed retractions and dynamic expansions. For example, f1d1, f3d3, and f4d4 cells have 68, 56, and 58% proteins, respectively, that repeat in the ranking of the first 100 created by the Bridging Centrality metric. Therefore, although a variation in the way of accounting for the conserved gene neighbourhood (fixed retraction versus a dynamic expansion) alters the topology of networks, most networks were observed to have significant similarities. These similarities also maintain high metric values that depend on the number of interactions/edges in an interaction network, as shown in Fig. 7.

**Table 5:** Metric values obtained for interaction networks by CN and PP

Id	CN expansion	Nodes	Medium degree	Density	Edges	Maximum degree
STRING	–	2213	180.83	0.082	200,088	901
f1	Fixed	2149	840.228	0.391	902,825	1316
f2	Fixed	2050	69.748	0.034	71,492	688
f3	Fixed	2057	270.628	0.132	278,341	689
f4	Fixed	1984	121.620	0.061	120,647	385
d1	Dynamic	2141	853.973	0.399	914,178	1355
d2	Dynamic	2045	91.318	0.045	93,373	705
d3	Dynamic	2045	289.11	0.141	295,615	772
d4	Dynamic	1976	132.947	0.067	131,356	469
d5	Dynamic	2058	272.208	0.132	280,102	713

## Discussion

Producing an interaction network is relatively simple; researchers simply need to find a reason to link pairs of entities and apply this rule for all possible pairs of a set. However, such a reason should be trustworthy, or else we could have messy, random, and ineffective relationships. Considerable time and resources could be lost in explaining a non-existent solution for an annotated relation among subjects. Thus, the fundamental role of always present and useful databases becomes clear. Some notable data sources for genome annotation include the following:

- Search Tool for the Retrieval of Interacting Genes/Proteins (STRING) [5];
- Database for Annotation, Visualization and Integrated Discovery (DAVID) [9];
- Metascape [10];
- Kyoto Encyclopedia of Genes and Genomes (KEGG) [11];
- Gene Ontology (GO) [12]; and
- Gene Expression Omnibus [13].

These well-known databases possess easy-to-use enrichment analyses and useful and user-friendly interfaces for biologists. Many of these databases allow researchers to export their results and continue additional studies using various programs, such as Python [14], Cytoscape [15], R [16], UALCAN [17], MCODE [18], and GEPHI [19]. Notably, there are a considerable number of libraries existing and deployed annually for all this software. For instance, such libraries enable researchers to focus on candidate hub genes, differentially expressed genes (DEGs), the tertiary structure of protein interactions, and many other useful features. For example, in [20], the authors studied crucial genes in hepatocellular cancer. The authors obtained the initial data from the Gene Expression Omnibus database. The DAVID website was employed to perform the GO and KEGG enrichment analyses before uploading the data to the STRING database, which was utilized for further analysing the DEGs. After that step, the authors used Cytoscape software to construct a protein interaction network. Once in Cytoscape, a plugin for MCODE was used to study the modules of DEGs. For a final analysis, the authors used the Gene Expression Profiling Interactive Analysis website to determine the module genes’ effects on overall survival under hepatocellular cancer. This research employs a notably elaborate combination of several databases and software tools to produce interesting in silico bioinformatic analyses. There are many other studies similar to this one [2]. Many of the cited databases in this section have the common characteristic of being sealed databases. We define sealed as not accepting new data from anyone outside a trained and specialized team of workers. There is nothing wrong with this approach; one does not allow others to access their bank accounts because of such concerns regarding unauthorized access. For instance, one cannot upload a new genome to the STRING database. First, the database administrator must ensure that the data are trustworthy. Second, a new genome should have some representativeness level to acquire a specific matching of annotation according to the genomes already in the database to reduce the risk of producing poor annotations. Nonetheless, many users would prefer to have their novel genomes annotated by such useful software. Indeed, users can upload their novel genomes to the STRING database and subject them to various kinds of enrichment according to a plethora of third-party databases but only for known genes, not for novel genes. A researcher investigating model organisms will not face such challenges in obtaining useful insights from all the databases mentioned earlier. For instance, when studying *H. sapiens, M. musculus, R. norvegicus, D. rerio, D. melanogaster, C. elegans, S. cerevisiae, A. thaliana, S. pombe,* and *P. falciparum*, if the STRING [5], Metascape [10], and DAVID [9] databases are employed, a list of genes is sufficient to provide useful data. However, when investigating unseen or underrepresented organisms, a researcher will not have a trustworthy list of genes. Many of the open reading frames (ORFs) will be of unknown function. Such a scenario is more likely to occur when studying prokaryotes. The study of prokaryotes yields dozens of novel genomes and thousands of novel genes daily. We believe that these novel data, even those not curated, deserve the benefit of doubt and further annotation, including topological annotations. We are also confident that the currently utilized databases will not easily manage such a massive volume of novel data. We support the parallelism of this considerable data novelty processing by the creators of the data, the researchers, not by centralized databases, at least in the early stages of data generation. To achieve our vision of parallelism, we developed GENPPI software. GENPPI transfers the question of topological annotation from the centralized databases to the final user, the researcher, at the initial point of research. GENPPI enables researchers to experiment among better sets of genomes to create topological annotation. For instance, we believe that the GENPPI topological annotation information is directly proportional to the number of genomes used to create an annotation. In contrast, the data are indirectly proportional to the number of genomes used for a GENPPI round. As we employ fewer genomes in an annotation round, GENPPI will suggest more interactions between the ORFs, since there are not too many genomes to confirm such a set of predictions as co-occurring. We constantly search for equity between data and information but are guided by the skills of researchers regarding the organisms under study. GENPPI inspects genomes represented as proteins in the multifasta format, searching for a conserved neighbourhood, phylogenetic profile, and gene fusion. This software enables the decision of how many and what genomes to use for the construction of a protein interaction network to be transferred to the final user. Despite the limited number of features employed in GENPPI, in the previous sections, we attempted to demonstrate that this set of characteristics suffices to produce good-quality networks. We attempted to support our hypotheses based on the construction of finely detailed phylogenetic maps for the genomes under study. We demonstrated that the features used by GENPPI can distinguish between, for example, the biovars of the species *Corynebacterium* *pseudotuberculosis* [21], as well as obtaining optimal separation among the genera of other prokaryotic organisms, although the software is not limited to unicellular organisms. Considering the quality of species separation and based on the three features analysed by GENPPI, our software obtained good quality for our topological annotations, as well as fewer computational resources needed for this task. For instance, for 50 genomes of an organism containing an of average 2200 genes, we spent only a matter of hours accomplishing full topological annotation.

### Why we are not comparing our results to STRING, directly

We sustain the quality of an interaction network based on the quality of the data used to create the relationships. We believe in the quality of an interaction network according to the potential of the data to describe known real-world connections. Considering we are using phylogenetic profile (PP) and conserved neighbourhood (CN) as the primary ground for interaction networks, we claim a trustable interaction network if CN and PP can separate genus, species, or subspecies. Depending on the implemented algorithm, one can have different interactions for the same set of genomes. However, even among unlikely software results, we can expect the correct ones to devise equivalent conclusions. For CN and PP, the derived interaction networks should correctly differentiate species and subspecies according to these features. Our research team had no access to CN and PP created by the software STRING concerning the genomes analyzed in this work. Such limitation does not allow us to compare our ground data to the STRING directly.

## Conclusions

The study of bacterial network topologies based on evolutionarily predicted relationships is a promising area of research. Until this study was conducted, few studies had performed such a query for a genome. A possible cause for this limitation is the absence of software to predict interaction networks from protein sequences alone. Our software presented in this report is a useful tool for any researcher to use. GENPPI can be another software tool for the scientific community to investigate many novel genomes constantly assembled. It would allow us to investigate noncore genes concerning the most known organisms, a more profound analysis with a particular species or a superficial one for unrelated species. Such a differentiated analysis is possible with GENPPI because it gives a researcher control over which ones and how many genomes we intend to use to answer a scientific question. We offer various configuration modes to employ, ranging from fast and lightweight to more careful and intense computations. However, we should warn users of the usual traps of extensive computational inquiries. Regardless of the chosen processing method, the user can be assured of obtaining a mostly reasonable answer, at least [22]. We are confident in the GENPPI software because the majority of the necessary relationships that it provided were determined to be correct by CN and PP, as phylogenetic analyses of these relations correctly separated bacterial species. Our software is open-source, and we can compile it for different operational systems.

## Availability and requirements

- Project name: genppi
- Project home page: genppi.facom.ufu.br
- Operating system(s): Platform independent
- Programming language: Common Lisp
- Other requirements: Not applicable
- License: GNU GPL
- Any restrictions to use by non-academics: licence needed

## Data Availability

We created a website for the genppi project accessible at “genppi.facom.ufu.br”. Moreover, we also created a GitHub project to handle the source code in the Common Lisp language, tools, binary files for different operating systems, and GENPPI software tutorials are available at “github.com/santosardr/genppi”.

## Abbreviations

CN: Conserved gene neighbourhood
PP: Phylogenetic profile
GF: Gene fusion
ANI: Average nucleotide identity
ORF: Open reading frame
STRING: Search Tool for the Retrieval of Interacting Genes/Proteins
